# Genetic basis of antigenic variation in foot-and-mouth disease serotype A viruses from the Middle East^☆^

**DOI:** 10.1016/j.vaccine.2013.08.102

**Published:** 2014-01-23

**Authors:** Sasmita Upadhyaya, Gelagay Ayelet, Guntram Paul, Donald P. King, David J. Paton, Mana Mahapatra

**Affiliations:** a The Pirbright Institute, Ash Road, Woking, Surrey, GU24 0NF, UK; b National Veterinary Institute, DebreZit, Ethiopia; c MSD Animal Health, Intervet International GmbH, Osterather Straße 1a, 50739 Cologne, Germany

**Keywords:** FMD virus, Antigenic variation, Capsid sequence, Epitopes, Polyclonal antibodies, Antigenic determinants

## Abstract

Foot-and-mouth disease viruses (FMDV) from serotype A exhibit high antigenic diversity. Within the Middle East, a strain called A-Iran-05 emerged in 2003, and subsequently replaced the A-Iran-96 and A-Iran-99 strains that were previously circulating in the region. Viruses from this strain did not serologically match with the established A/Iran/96 vaccine, although most early samples matched with the older A22/Iraq vaccine. However, many viruses from this strain collected after 2006 had poor serological match with the A22/Iraq vaccine necessitating the development of a new vaccine strain (A/TUR/2006). More recently, viruses from the region now exhibit lower cross-reactivity with the A/TUR/2006 antisera highlighting the inadequacy of the serotype A vaccines used in the region. In order to understand the genetic basis of these antigenic phenotypes, we have determined the full capsid sequence for 57 Middle Eastern viruses isolated between 1996 and 2011 and analysed these data in context of antigenic relationship (*r*_1_) values that were generated using antisera to A22/Iraq and A/TUR/2006. Comparisons of capsid sequences identified substitutions in neutralising antigenic sites (1, 2 and 4), which either individually or together underpin these observed antigenic phenotypes.

## Highlights

• The recent A-Iran-05 viruses circulating in middle East do not match with the existing vaccine strains.
• We have generated full capsid sequence of 51 A-Iran-05 viruses and their corresponding antigenic (serological) relationship (*r*_1_) values using antisera raised against A22 and A/TUR/2006 vaccine.
• Analysis of the data to correlate genotype to antigenic phenotype revealed amino acid changes in neutralising antigenic sites.

## 1 Introduction

Foot-and-mouth disease (FMD) remains a globally important livestock disease affecting cloven-hoofed animals. It remains enzootic in many regions, especially in developing countries where it imposes a trade barrier upon livestock and their products. The causative agent, FMD virus (FMDV) has a rapid mutation rate and exists in seven immunologically distinct serotypes, O, A, C, Asia 1, SAT (Southern African Territories) 1, 2 and 3, each with a wide spectrum of antigenically distinct subtypes.

FMDV is a single-stranded, positive-sense RNA virus (Genus *Aphthovirus*, family *Picornaviridae*). The viral genome is about 8.3 kb long, enclosed within a protein capsid. The capsid is composed of 60 copies each of four different structural proteins (VP1-4); VP1-3 are surface exposed while VP4 is entirely internal. Crystallographic studies have identified the structure of the FMDV capsid [1], [2] and immunological epitopes have been mostly found on surface-oriented interconnecting loops between structural elements. Studies employing monoclonal antibodies (mAb) have identified antigenic sites by sequencing mAb neutralisation resistant (mar) mutants [3], [4], [5], [6], [7], [8], [9]. Of the five antigenic sites reported so far for the most extensively studied serotype O, site-1 (G-H loop) is linear and trypsin-sensitive whereas the others are conformational and trypsin-resistant. Equivalent neutralising antigenic sites (except site 3) have also been identified for serotype A, with critical residues present in equivalent positions [3], [4], [5], [6], [9].

Serotype A viruses are present on all continents where FMD is reported, and is antigenically diverse [10] often exhibiting poor cross-protection [11]. In the Middle East (ME), a new variant, A-Iran-05, was identified in samples collected from Iran in 2003 and subsequently spread to neighbouring countries [10] and North Africa [12]. This genotype replaced the A-Iran-96 and A-Iran-99 genotypes that were previously circulating in the region; did not cross-react with A/Iran/96 vaccine antisera and shared a closer antigenic relationship with the older A22/Iraq vaccine strain (v/s) [10]. However, many samples isolated after 2006 did not even match with A22/Iraq v/s and so a new v/s, A/TUR/2006 was introduced. From sequence data, Jamal and colleagues indicated candidate amino acid (aa) substitutions in the capsid that might have contributed to these antigenic changes [13]. More recently, there is evidence that viruses from the region now exhibit lower cross-reactivity with the A/TUR/2006 antisera. The aim of this study was to investigate the molecular basis of the antigenic variation in these viruses using capsid sequences and their corresponding antigenic relationship (*r*_1_) values.

## 2 Materials and methods

### 2.1 Cells and viruses

Fifty-seven serotype A viruses from the ME submitted to the Food and Agriculture Organisation's World Reference Laboratory for FMD (WRLFMD) at the Pirbright Institute were used in this study (Supplementary table). Two are the v/s A22/IRQ/24/64 (A22/Iraq) and A/TUR/2006 that were originally isolated in Iraq and Turkey, in 1964 and 2006 respectively; the 55 other viruses were isolated over a fifteen year period (1996–2011). These viruses were from seven ME countries, Turkey (*n* = 17), Iran (*n* = 26), Iraq (*n* = 2), Pakistan (*n* = 5), Afghanistan (*n* = 4), Saudi Arabia (*n* = 1) and Jordan (*n* = 2) (Supplementary table). These samples were derived from cattle epithelial tissues (except one of ovine origin), and were initially grown in primary bovine thyroid cells with subsequent passage in either BHK-21 or IB-RS2 cells. Stocks of virus were prepared by infecting IB-RS2 cell monolayers and were stored as clarified tissue culture harvest at −70 °C until required.

Supplementary data associated with this article can be found, in the online version, at http://dx.doi.org/10.1016/j.vaccine.2013.08.102.

Supplementary Table S1 List of serotype A viruses used in this study. nd: not designated; nk: not known. The P1 sequences have been submitted to Gene Bank and awaiting accession numbers.

### 2.2 Polyclonal serum

Antisera were prepared against serotype A FMD viruses (A22/Iraq and A/TUR/2006) by immunising five cattle per v/s with inactivated, purified 146S FMD virus particles in ISA-206 adjuvant. Bulk blood was collected on 21 day post-vaccination for preparation of sera. For each antigen, a pool of sera from five animals was used in the serological tests. The A22/Iraq and A/TUR/2006 antisera exhibited equivalent homologous titres (log_10_ 2.43 and 2.54, respectively) by virus neutralisation test (VNT).

### 2.3 Two-dimensional micro neutralisation assay (2D-VNT)

The 2D-VNT was carried out using the 21-day post-vaccination sera following established methodology [14]. Antibody titres were calculated from regression data as the log_10_ reciprocal antibody dilution required for 50% neutralisation of 100 tissue culture infective units of virus (log_10_SN_50_/100 TCID_50_). The antigenic relationship of viruses based on their neutralisation by antibodies is given by the ratio: ‘*r*_1_′ = neutralising antibody titre against the heterologous virus/neutralising antibody titre against the homologous virus. Differences in the *r*_1_-values obtained by the polyclonal antiserum were evaluated according to standard criteria [15].

### 2.4 Nucleotide (nt) sequencing and analysis of the sequence data

The sequences of the entire capsid coding region (P1) of selected viruses were generated. RNA extraction from the cell culture grown viruses and reverse transcription (RT) were performed as described [16]. PCR was carried out using the “KOD hot-start DNA polymerase” kit (Novagen) as recommended by the manufacturer, using the forward primer L463F (5′-ACCTCCRACGGGTGGTACGC-3′) and one of the reverse primers NK72 (5′-GAAGGGCCCAGGGTTGGACTC-3′) or EUR2B52R (5′-GACATGTCCTCCTGCATCTGGTTGAT-3′). PCR products were purified using the QIAquick PCR purification kit (Qiagen) according to the manufacturer's instructions and sequenced using BigDye^®^ Terminator v3.1 Cycle Sequencing Kit (Applied Biosystems, Carlsbad, CA, USA) using the PCR primers and additional internal sequencing primers (sequences available on request). Sequences (from the ABI 3730 machine) were assembled and analysed using SeqMan II (DNAStar Lasergene 8.0). Nucleotide sequences of the viruses were aligned using the CLUSTAL X multiple sequence alignment program [17] and the predicted aa sequences were translated using BioEdit 7.0.1 [18]. Alignments were used to construct distance matrices using the Kimura 2-parameter nucleotide substitution model [19] as implemented in the programme MEGA 4.0 [20].

### 2.5 Bayesian phylogenetic analysis

The complete P1 sequence of the viruses belonging to the A-Iran-05 strain (*n* = 51) were aligned and subjected to jModelTest 0.1.1 [21]. The general time reversible (GTR) model for substitution model with combination of gamma distribution and proportion of invariant sites (GTR + I + G) was found to be the best model for the Bayesian analysis of the sequence dataset. Analysis was performed using the BEAST software package v1.5.4 [22] with the maximum clade credibility (MCC) phylogenetic tree inferred from the Bayesian Markov Chain Monte Carlo (MCMC) method. The age of the viruses were defined as the date of sample collection. In BEAUti v1.5.4, the analysis utilised the GTR + I + G model to describe rate heterogeneity among sites. In order to accommodate variation in substitution rate among branches, a random local clock model was chosen for this analysis [23]. BEAST output was viewed with TRACER 1.5 and evolutionary trees were generated in the FigTree program v1.3.1.

### 2.6 Data analysis

The proportion of synonymous substitutions per potential synonymous site and the proportion of non-synonymous substitutions per potential non-synonymous site were calculated by the method of Nei and Gojobori [24] using the SNAP program (www.hiv.lanl.gov). The aa variability of the capsid region of the A-Iran-05 viruses was determined as described by Valdar [25]. Statistical analyses used Minitab release 12.21 software.

## 3 Results and discussion

The A-Iran-05 viruses, first detected in Iran [10], spread to neighbouring countries in the ME [10], [12], [13], and spawned sub-lineages over the next seven years. Most sub-lineages died out, whereas a few persisted and became dominant, and some are still circulating. In this study, we have focussed mainly on three sub-lineages, namely ARD-07, AFG-07 and BAR-08. ARD-07, first detected in Ardahan, Turkey in August 2007 was the main circulating strain in Turkey during 2007–2010. However, it has not been detected in samples received in WRLFMD, Pirbright from Turkey during 2011–2012. AFG-07, first isolated from a bovine sample in Afghanistan in 2007 has spread to other neighbouring countries such as Bahrain, Iran, Pakistan and Turkey. BAR-08, first detected in a bovine sample in the Manama region of Bahrain in 2008 has spread to other countries such as Iran, Pakistan and Turkey. This sub-lineage has also jumped to North African countries, such as Libya in 2009 [12] and Egypt in 2010 and 2011 (http://www.wrlfmd.org), probably because of trade links with ME countries. Evolution of the serotype A viruses in the ME has resulted in the appearance of further sub-lineages like HER-10 and SIS-10. These sub-lineages have gained dominance over the others and have been reported to be actively circulating in this region in years 2011 and 2012 (http://www.wrlfmd.org).

### 3.1 Serological characterisation of the type A viruses

**Fig. 1:**
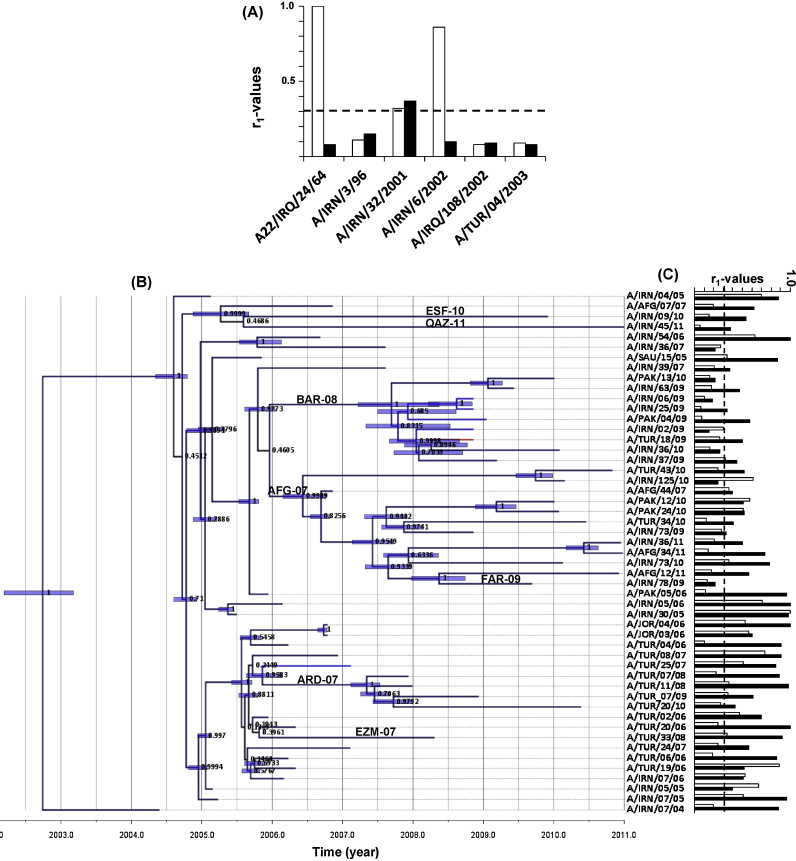
(A) Antigenic relationship (*r*_1_) values of pre-A-Iran-05 viruses. A22/Iraq (white bars) and A/TUR/2006 (black bars). The horizontal dotted line indicates the cut-off value of 0.3, above which the vaccine is considered to be a good match. (B) Bayesian phylogenetic tree of the A-Iran-05 viruses and respective *r*_1_-values against A22/Iraq (white bars) and A/TUR/2006 (black bars) antisera. The sub-lineages defined by WRLFMD on the basis of VP1 sequences are labelled on the respective branches.

The cross-reactivity of the type A viruses from the ME were measured by 2D-VNT using A22/Iraq and A/TUR/2006 post-vaccination sera. The six pre-2004 viruses (A-Iran-96/non-designated strains) exhibited weak cross-reactivity with these antisera except A22/Iraq and A/IRN/6/2002 that cross-reacted with the A22/Iraq antisera (Fig. 1A). For the A-Iran-05 strain, viruses isolated in early years reacted well with A22/Iraq anti-sera, whereas isolates after 2006 exhibited lower reactivity (Fig. 1C). Most of these viruses exhibited higher cross-reactivity with the newer A/TUR/2006 vaccine antisera. However, viruses from Iran, Pakistan and Turkey belonging to sub-lineages BAR-08 and ARD-07 exhibited lower cross-reactivity with the A/TUR/2006 antisera (Fig. 1C).

### 3.2 Genetic characterisation of the type A viruses

The complete capsid sequence of 57 serotype A viruses generated in this study were 2205 nt long except A/IRQ/108/2002 (A-Iran-96 strain) that had a 3-nt deletion at position 1984–1986 of P1, resulting in deletion of an aa at position VP1-138 in the G–H loop which has been reported to be a dominant antigenic site [4]. When compared to the sequence of the A22/Iraq v/s there was 17.0–20.6% nt variation between these viruses: A/IRN/03/96 sharing the closest nt identity and A/IRN/45/2011 being the most variable. Analysis of the capsid aa sequences revealed 6.1–18.1% variation, A/IRN/30/2005 and A/IRN/05/2006 having the closest, and A/IRN/45/2011 having the lowest aa identity, respectively. Similarly, when compared to the capsid sequence of the A/TUR/2006 v/s, the nt variability was found to vary from 0.8 (A/TUR/02/2006) to 19.3% (A/TUR/04/2003) with a 0.5 (A/IRN/07/2006) to 9.1% (A/TUR/04/2003) variation at the aa level.

*Phylogenetic analysis* of the capsid sequences revealed all the viruses belong to the ASIA topotype within serotype A FMDV. The viruses isolated from 2004 onwards formed a new genetic strain, A-Iran-05, distinct from previous virus strains reported to be present in the region, similar to an earlier report [10]. Various sub-lineages within the A-Iran-05 strain have been defined based on the analysis of VP1 sequences. The samples used in this study included 9 samples from BAR-08, 11 from AFG-07, 4 from ARD-07 and one each from ESF-10, FAR-09, QAZ-11 and EZM-07 (Supplementary table). The sub-lineages, BAR-08 and AFG-07 shared a common ancestor which evolved into two distinct sub-lineages over time, whereas most of the contemporary viruses gradually died out. A/IRN/78/2009 belongs to sub-lineage FAR-09 that has evolved from the AFG-07 sub-lineage, and is currently circulating in the region. A/AFG/12/2011 has not been assigned a sub-lineage yet, however, shares a common ancestor (AFG-07 sub-lineage) with A/IRN/78/2009. This pattern is also consistent with that observed when phylogenetic trees are drawn using only VP1 sequences (data not shown). Additional phylogenetic analysis of seven A-Iran-05 isolates from Pakistan and Afghanistan [13] revealed that the isolates belonging to AFG-07 or BAR-08 sub-lineages cluster with sequences of viruses from the same sub-lineage used in this study (data not shown).

### 3.3 Rate of nucleotide substitution per site

From the BEAST analysis using a random local molecular clock, the rate of substitution of all the nt changes in the capsid coding region of the A-Iran-05 viruses was estimated to be 1.06 × 10^−2^/site/year (95% HPD 9.53 × 10^−3^ to 1.05 × 10^−2^). This is similar to the report (1.12 × 10^−2^/site/year) for VP1 sequences of A-Iran-05 viruses [13]; but higher than those reported by others [26], [27], [28], [29], [30], [31], [32]. The high evolutionary rate of serotype A viruses in the ME is resulting in emergence of new variants in the region.

### 3.4 A-Iran-05 viruses

An unbiased analysis of capsid sequences of the 51 A-Iran-05 viruses revealed 692 nt substitutions at 637 sites distributed across the region (Fig. 1B). Out of these, 80.05% of nt substitutions were found to be synonymous (silent) and 19.95% were non-synonymous (non-silent). Forty seven sites were identified to have been substituted twice and four were substituted three times. At one site (VP2-134) the first two bases of the codon were mutated encoding 5 different aa (P->T/S/L/H). This residue is located very close to residues VP2-132 and 133 that were reported as critical by mar-mutant studies for A10 virus [9]. In addition, the residue at this position has been reported to strongly influence the binding of antigenic site-2 mAbs in serotype O viruses [16]. Out of the four sites with three nt substitutions (encoding 2–4 aa residues), three were present in VP3 and one in VP1 (Table 1A).

**Table 1A:** Capsid nucleotide positions with three nucleotide substitutions and associated amino acid changes relative to A/IRN/07/2004 virus. The number of isolates in which these changes were observed are shown in parenthesis.

Capsid nucleotide position	Codon position	Amino acid substitutions
1119	VP3 70	D → A(2)/E(7)/N(1)
1305	VP3 132	T → N(16)/A(1)
1521	VP3 204	V → A(1)
1656	VP1 28	Q → H(20)

**Fig. 2:**
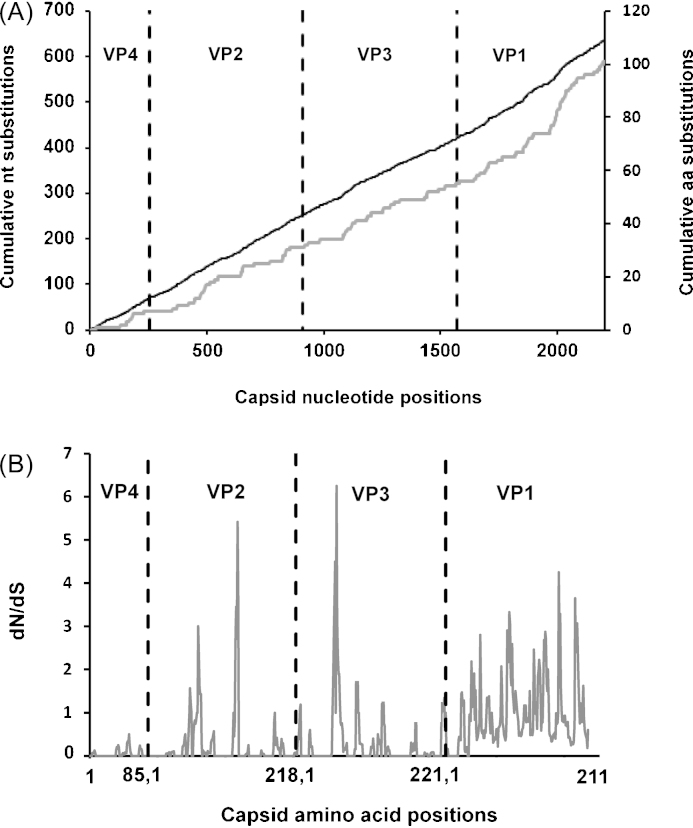
(A) Cumulative changes in capsid coding regions of A-Iran-05 viruses. The black line indicates total cumulative nucleotide changes, the grey line, cumulative amino acid changes. (B) dN/dS of capsid of A-Iran-05 viruses. The vertical black dotted line indicates gene junctions.

The analysis of the capsid aa residues of A-Iran-05 viruses revealed 140 substitutions at 101 sites across the capsid (Fig. 2A) with some sites having 2–5 alternate aa (Table 1B). Interestingly, sequences for VP1-204 encoded five different aa and exhibited nt changes at all the three positions within the codon as did VP1-196, with changes at all the three positions of the codon giving rise to four alternative aa. In addition, the non-synonymous nt substitutions were not equally distributed across the capsid coding regions: there were several local areas where the dN/dS ratio was higher than in other parts of the sequence alignment (Fig. 2B). One region in VP3 (57–65), two in VP2 (75–76 and 130–134) and eight regions in VP1 (52–53, 83–84, 92–105, 131–132, 137–141, 145–152, 168–171 and 192–204) had dN/dS ratio of >1 indicative of sites under strong positive selection.

**Table 1B:** Capsid positions where multiple amino acid substitutions were observed. The number of isolates in which these changes were observed are shown in parenthesis.

Viral protein	Positions with three alternative amino acid residues	Positions with four alternative amino acid residues	Positions with five alternative amino acid residues
VP4	77 (2)	–	–

VP2	39 (2), 56 (3), 79 (9)	–	134 (18)

VP3	8 (17), 25 (3), 59 (12)	70 (10)	–
	61 (2), 132 (17), 139 (3)		
	175 (3), 220 (22)		

VP1	4 (2), 45 (14), 108 (4)	24 (4), 43 (23), 96 (16)	204 (6)
	139 (2), 140 (5), 149 (23)	99 (12), 141 (27), 196 (22)	–
	154 (2), 171 (3)		

### 3.5 Amino acid variability of the capsid of the A-Iran-05 viruses

**Fig. 3:**
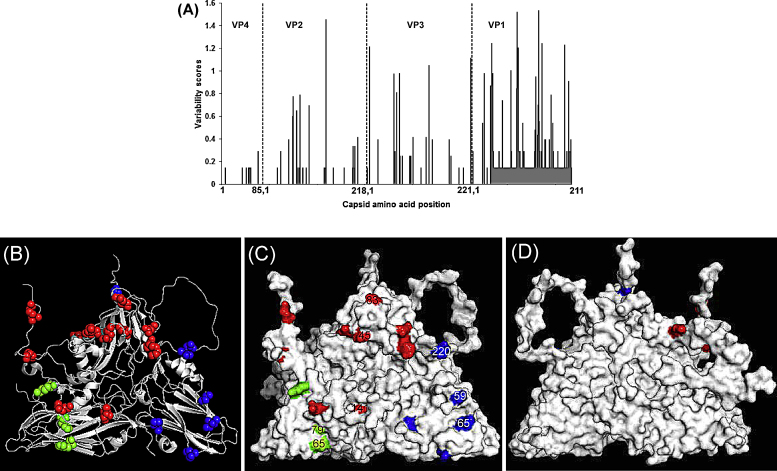
(A) Capsid amino acid variability of A-Iran-05 viruses. The vertical black dotted line indicates gene junctions. (B–D) 3D structure of Type A protomer, modelled using O1 BFS co-ordinates (1FOD, reduced) [2] with highly variable type A capsid amino acid residues (with a score of more than 0.75) highlighted. VP1 residues (red), VP3 (blue), VP2 (green); (B) cartoon; (C) external surface; (D) internal surface. (For interpretation of the references to colour in this figure legend, the reader is referred to the web version of the article.)

Investigation of aa variability across the capsid of the A-Iran-05 viruses revealed VP4 to be highly conserved and VP1 least conserved (Fig. 3A); similar to an earlier report [13]. The residues with a score greater than 0.75 (3 in VP2, 6 in VP3 and 12 in VP1) are shown in Fig. 3B-D indicating that over 50% of the residues with very high variability scores were present in VP1 (Fig. 3A). All these residues were found to be surface-exposed, except one residue in the N-terminus of VP1 (position 28) and one in N-terminus of VP3 (position 8) (Fig. 3C and D).

### 3.6 Correlating genotype to antigenic phenotype

A number of aa changes were observed between the v/s and field isolates, however no linear correlations between the *r*_1_-values and the total number of aa changes in the capsid encoding regions were observed (data not shown). When compared to the A22/Iraq vaccine, these viruses had more than 40 aa changes in the capsid region, whilst about 35% of these had *r*_1_ values above 0.3 indicating a good match. This indicates that a large proportion of the substitutions are neutral and only a few, located at particular capsid positions impact on the antigenic nature of the virus. Similar analyses were also carried out to study if the *r*_1_-values correlated with the number of aa changes in each of the individual structural proteins (VP1-4); however no linear correlation was observed (data not shown).

### 3.7 Comparison between A22/Iraq, A/TUR/2006 v/s and BAR-08 field isolates

**Fig. 4:**
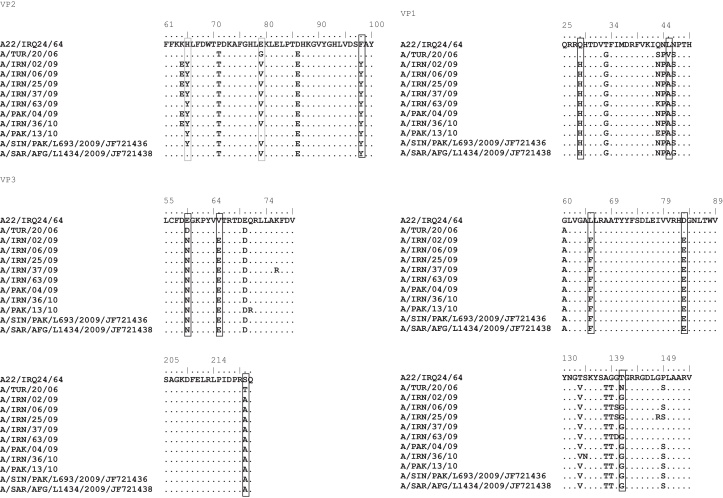
Capsid amino acid substitution in BAR 08 viruses compared to A22/Iraq and A/TUR/2006 v/s. The sequences of two isolates (A/SIN/PAK/L693/2009/JF721436 and A/SAR/AFG/L1434/2009/JF721438) were extracted from GenBank. The residues with the change are boxed. The grey box in VP2 indicates substitutions in nine out of 10 viruses.

**Fig. 5:**
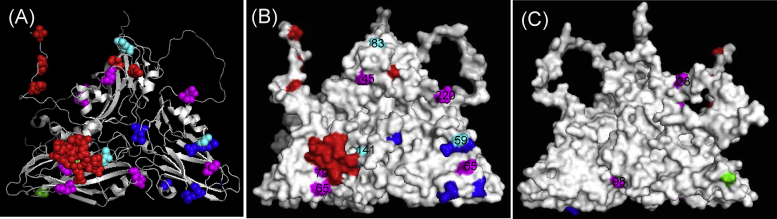
3D structure of a type A protomer, modelled using O1 BFS co-ordinates (1FOD, reduced) [2] with type A residues highlighted. The critical amino acid residues that have been identified by mar mutant studies are highlighted; VP1 residues (red), VP2 (green), VP3 (blue); aa residues identified in BAR-08 viruses (magenta), aa residues identified in BAR-08 viruses that has also been identified by mar-mutant studies (cyan). (A) Cartoon; (B) External surface; (C) Internal surface. (For interpretation of the references to colour in this figure legend, the reader is referred to the web version of the article.)

*In vitro* testing of viruses belonging to the BAR-08 sub-lineage with either A22/Iraq or A/TUR/2006 antisera generated low *r*_1_-values indicating lower expected protection. The capsid aa sequences of these viruses, including sequences for two isolates previously reported [13], were analysed further to understand the changes in the antigenicity of these viruses. As most of these viruses do not cross-react with the antisera of either of the v/s, we specifically looked for aa residues in the field isolates which were different from those of both the v/s (Fig. 4). A total of 11 aa residues were identified; three residues (VP1-45, 65 and VP3-59) were indicated in a similar study [13]. Three residues were eliminated as being either completely (VP1-28) or partly (VP2-98) on the internal surface of the virion (Fig. 5C), or completely (VP1-65) buried in the structure; though Jamal and colleagues indicated substitution of VP1-65 may change the surface structure [13]. The remaining eight residues (VP1-45, 83, 141; VP2-65, 79; VP3-59, 65, 220) were surface-exposed (Fig. 5B) and are therefore good candidates to explain the inability of the antisera to cross-react with the field isolates. The substitutions in VP2-65 and 79 were recorded in nine out of 10 isolates studied. We excluded VP1-45 because (i) both the residues are hydrophobic; (ii) this/adjacent residues were reported to be part of antigenic site-3 in case of serotype O viruses [7] and SAT 1 [33], however this has never been reported in serotype A mar-mutant studies; (iii) this residue is also picked up by epitope prediction software, however, mutation of this residue in a cDNA clone did not have much impact on the antigenicity of the virus (F. Bari and M. Mahapatra, unpublished results). Three residues VP1-83, 141 and VP3-59 (shown in cyan in Fig. 5B) have been reported to be critical in serotype A mar-mutant studies [3], [4], [5], [9]. A change in these residues may affect the overall conformation of the viral capsid and thereby alter the antigenicity of the virus. VP3-220 is located in close proximity to the C-terminus of VP1 of an adjacent protomer, and in close vicinity to residue VP3-218, which was recently reported to be critical in serotype Asia 1 [8]. In addition, all these residues were highly variable among the A-Iran-05 viruses (Fig. 2) and four of these, namely VP1-83, 141, VP3-59 and 65 exhibited very high dN/dS values (2, 2, 4.5 and 1.9, respectively) indicating strong positive selection.

### 3.8 Comparison between A/TUR/2006 v/s and ARD-07 field isolates

The four serotype A viruses (isolated from Turkey) of ARD-07 sub-lineage were found to cross-react with the A/TUR/2006 v/s. However, two recent viruses (A/TUR/7/2009 and A/TUR/20/2010) exhibited comparatively lower reactivity with these antisera. The capsid aa sequence of these four viruses along with that of the v/s were aligned and analysed further leading to the identification of two residues, VP1-24 (A-V) and VP2-70 (D-E). VP1-24 is internal, whereas VP2-70 is present on the outer surface of the capsid (data not shown). In case of A5 virus, adjacent residues like VP2-72 (D-N) and 79 (Q-G/V) have been reported to be critical for mAb binding [6]. Moreover VP2-70 has been reported to be critical in neutralising antigenic site 2 of serotype O viruses [7]. In addition, epitopes present in this area have recently been reported to be dominant within the polyclonal response of serotype O vaccinated animals and mutations in this area resulted in significant reduction in neutralising antibody titres [34].

In summary, analysis of serology and capsid sequence data of BAR-08 and ARD-07 viruses revealed aa changes involving neutralising antigenic sites 1, 2 and 4 of serotype A viruses that could be responsible for the antigenic variation in these viruses. Targeted mutagenesis studies involving a cDNA clone could confirm these observations. A consequence of the high rate of evolution in FMDV and emergence of new sub-lineages of serotype A viruses, the ME has required the regular development of new v/s typically every 5–10 years. Therefore, close monitoring of the outbreak strains in the region is essential to enable appropriate vaccines to be selected for use in FMD control programmes; and the need to develop a new v/s should be identified in a timely fashion to prevent future outbreaks. In such situations where the match between v/s(s) and circulating field viruses is suboptimal, other steps that improve population immunity become especially important, such as ensuring the quality and potency of the vaccines; correct targeting and coverage of vaccines; the use of booster doses in a timely manner, especially in young animals and those susceptible livestock that are likely to be traded.
