# Supplementary material for: Genetic basis of antigenic variation in foot-and-mouth disease serotype A viruses from the Middle East
Source: Vaccine. 2014 Jan 23;32(5):631–8. doi: 10.1016/j.vaccine.2013.08.102 (PMC3898079; doi:10.1016/j.vaccine.2013.08.102)
Supplement: Supplementary Table S1 — List of serotype A viruses used in this study. nd: not designated; nk: not known. The P1 sequences have been submitted to Gene Bank and awaiting accession numbers. [file mmc1.pdf]

Supplementary table – List of serotype A viruses used in this study. nd: not designated, nk: not known. The P1 sequences have been submitted to Gene Bank and awaiting accession numbers.

No.	WRL no.	Topotype	Strain	Sub-lineage	Geographic origin (Province, country)	Host species	Date collected	Accession No.
1	A_{22}/IRQ/24/64	ASIA	A_{22}	nd	Mosul, Iraq	Bovine	1964	submitted
2	A/IRN/3/96	ASIA	Iran-96	nd	Tehran, Iran	Bovine	03/11/1996	submitted
3	A/IRN/32/2001	ASIA	nd	nd	Tehran, Iran	Bovine	03/09/2001	submitted
4	A/IRN/6/2002	ASIA	nd	nd	Qom, Iran	Bovine	30/12/2002	submitted
5	A/IRQ/108/2002	ASIA	Iran-96	nd	Erbil, Iraq	Bovine	Oct-02	submitted
6	A/TUR/4/2003	ASIA	Iran-96	nd	Erzurum, Turkey	Bovine	2003	submitted
7	A/IRN/7/2004	ASIA	Iran-05	nd	Sistan and Baluchestan, Iran	Bovine	16/07/2004	submitted
8	A/IRN/4/2005	ASIA	Iran-05	nd	Qom, Iran	nk	11/04/2005	submitted
9	A/IRN/5/2005	ASIA	Iran-05	nd	Talkhqab, Markari, Iran	Bovine	20/04/2005	submitted
10	A/IRN/7/2005	ASIA	Iran-05	nd	Fars, Iran	Ovine	15/05/2005	submitted
11	A/IRN/30/2005	ASIA	Iran-05	nd	Qom, Iran	Bovine	21/08/2005	submitted
12	A/SAU/15/2005	ASIA	Iran-05	nd	Nr Riyadh, Saudi Arabia	Bovine	27/12/2005	submitted
13	A/JOR/3/2006	ASIA	Iran-05	nd	Mugar, Jordan	nk	06/12/2006	submitted
14	A/JOR/4/2006	ASIA	Iran-05	nd	Mugar, Jordan	nk	06/12/2006	submitted
15	A/IRN/5/2006	ASIA	Iran-05	nd	Tehran, Iran	Bovine	18/04/2006	submitted
16	A/IRN/7/2006	ASIA	Iran-05	nd	Esfahan, Iran	Bovine	23/04/2006	submitted
17	A/IRN/54/2006	ASIA	Iran-05	nd	Isfahan, Iran	Bovine	Nov-2006	submitted
18	A/PAK/5/2006	ASIA	Iran-05	nd	Karachi, Pakistan	Bovine	31/01/2006	submitted
19	A/TUR/2/2006	ASIA	Iran-05	nd	Kirklareli, Turkey	Bovine	01/02/2006	submitted
20	A/TUR/4/2006	ASIA	Iran-05	nd	Kars province, Turkey	Bovine	15/05/2006	submitted
21	A/TUR/6/2006	ASIA	Iran-05	nd	Samsun, Turkey	Bovine	17/05/2006	submitted
22	A/TUR/19/2006	ASIA	Iran-05	nd	Kastamonu, Turkey	Bovine	22/06/2006	submitted
23	A/TUR/20/2006	ASIA	Iran-05	nd	Eskisehir, Turkey	Bovine	23/06/2006	submitted
24	A/AFG/7/2007	ASIA	Iran-05	nd	<not known>, Afghanistan	Bovine	2007	submitted
25	A/AFG/44/2007	ASIA	Iran-05	AFG-07	<not known>, Afghanistan	Bovine	2007	submitted
26	A/IRN/36/2007	ASIA	Iran-05	nd	Sistan and Baluchestan, Iran	Bovine	Oct-2007	submitted
27	A/IRN/39/2007	ASIA	Iran-05	nd	Sistan and Baluchestan, Iran	Bovine	Oct-2007	submitted
28	A/TUR/8/2007	ASIA	Iran-05	nd	Malatya, Turkey	Bovine	01/02/2007	submitted
29	A/TUR/24/2007	ASIA	Iran-05	nd	Corum, Turkey	Bovine	02/04/2007	submitted

30	A/TUR/25/2007	ASIA	Iran-05	nd	Mus, Turkey	Bovine	06/04/2007	submitted
31	A/TUR/7/2008	ASIA	Iran-05	ARD-07	Marmara, Turkey	Bovine	31/01/2008	submitted
32	A/TUR/11/2008	ASIA	Iran-05	ARD-07	Inner Anatolia, Turkey	Bovine	16/02/2008	submitted
33	A/TUR/33/2008	ASIA	Iran-05	EZM-07	Inner Anatolia, Turkey	Bovine	12/06/2008	submitted
34	A/IRN/2/2009	ASIA	Iran-05	BAR-08	West Azerbaijan, Iran	nk	01/01/2009	submitted
35	A/IRN/6/2009	ASIA	Iran-05	BAR-08	Khorasan Razavi, Iran	nk	01/01/2009	submitted
36	A/IRN/25/2009	ASIA	Iran-05	BAR-08	Kerman, Iran	nk	01/01/2009	submitted
37	A/IRN/37/2009	ASIA	Iran-05	BAR-08	Kordestan,Iran	Bovine	06/05/2009	submitted
38	A/IRN/63/2009	ASIA	Iran-05	BAR-08	Qom, Iran	Bovine	02/08/2009	submitted
39	A/IRN/73/2009	ASIA	Iran-05	AFG-07	Kermah, Iran	nk	01/01/2009	submitted
40	A/IRN/78/2009	ASIA	Iran-05	FAR-09	Fars, Iran	nk	04/11/2009	submitted
41	A/PAK/4/2009	ASIA	Iran-05	BAR-08	Punjab, Pakistan	Bovine	12/03/2009	submitted
42	A/TUR/7/2009	ASIA	Iran-05	ARD-07	Black Sea, Turkey	Bovine	01/02/2009	submitted
43	A/TUR/18/2009	ASIA	Iran-05	BAR-08	<not known>, Turkey	Bovine	2009	submitted
44	A/IRN/9/2010	ASIA	Iran-05	ESF-10	Esfahan, Iran	Bovine	23/01/2010	submitted
45	A/IRN/36/2010	ASIA	Iran-05	BAR-08	West Azerbaijan, Iran	Bovine	27/03/2010	submitted
46	A/IRN/73/2010	ASIA	Iran-05	AFG-07	Gilan, Iran	Bovine	09/04/2010	submitted
47	A/IRN/125/2010	ASIA	Iran-05	AFG-07	Sistan and Baluchistan, Iran	Bovine	21/04/2010	submitted
48	A/PAK/12/2010	ASIA	Iran-05	AFG-07	Khushab, Pakistan	Buffalo	26/02/2010	submitted
49	A/PAK/13/2010	ASIA	Iran-06	BAR-08	Khushab, Pakistan	Buffalo	26/02/2010	submitted
50	A/PAK/24/2010	ASIA	Iran-05	AFG-07	Taxilla, Pakistan	Buffalo	22/03/2010	submitted
51	A/TUR/20/2010	ASIA	Iran-05	ARD-07	Black Sea, Turkey	Bovine	13/07/2010	submitted
52	A/TUR/34/2010	ASIA	Iran-05	AFG-07	East Anatolia, Turkey	Bovine	13/08/2010	submitted
53	A/TUR/43/2010	ASIA	Iran-05	AFG-07	Aegean An, Turkey	Bovine	26/12/2010	submitted
54	A/AFG/12/2011	ASIA	Iran-05	nd	Logar, Afghanistan	nk	28/01/2011	submitted
55	A/AFG/34/2011	ASIA	Iran-05	AFG-07	Jwzjan, Afghanistan	nk	16/02/2011	submitted
56	A/IRN/36/2011	ASIA	Iran-05	AFG-07	Kermanshah, Iran	Bovine	07/02/2011	submitted
57	A/IRN/45/2011	ASIA	Iran-05	QAZ-11	East Azar, Iran	Bovine	20/02/2011	submitted
